# Supplementary material for: WordCommentsAnalyzer: A windows software tool for qualitative research
Source: F1000Res. 2018 Sep 4;7:536. Originally published 2018 May 3. [Version 2] doi: 10.12688/f1000research.14819.2 (PMC6137411; doi:10.12688/f1000research.14819.2)
Supplement: Supplementary file 3 [file f1000research-7-17614-s0002.tgz › b2a79496-4ada-47c4-9682-a99ce867836d.zip!/Eight Iranian Health Journals/Yafteh _5.docx]

© Yafteh  . This work is licensed under CC BY 4.0 (https://creativecommons.org/licenses/by/4.0/).

Volume & Issue: دوره 19 - شماره 1

Title: بررسی جهش‌های ژن AR در زنان مبتلا به ناباروری

http://yafte.lums.ac.ir/article-1-2473-fa.html

مقدمه: ناباروری یک بیماری چند عاملی است. تغییرات و اختلالات هورمونی و ژنتیکی از جمله عوامل مهم در ناباروری زنان محسوب می‌شوند. تکوین و بلوغ تخمک‌گذاری وابسته به مسیرهای پیام‌رسانی مولکولی پاسخ دهنده به آندروژن‌ها است. بیش از صدها جهش منجر به مقاومت در عملکرد ژن گیرنده آندروژن (AR) ثبت گردیده است که از بین آنها می‌توان به ناحیه پلی‌مورفیک 5’UTR اشاره نمود. لذا با توجه به نقش گیرنده آندروژن در ناباروری، این مطالعه باهدف بررسی جهش‌های ژن AR در ناباروری زنان ایرانی انجام گردید. مواد و روش ­ها: در این مطالعه از 50 زن نابارور و 80 زن سالم به‌عنوان کنترل، نمونه خون تهیه شد. بعد از استخراج DNA، جهت تعیین جهش‌های ژن AR روش PCR مورداستفاده قرار گرفت. یافته­ ها: در مطالعه حاضر در ناحیه 5’UTR ژن گیرنده آندروژن یک حذف نوکلئوتید T در موقعیت 25+ مشاهده شد که این جهش تک نوکلئوتیدی باعث تغییر در بیان ژن گیرنده آندروژن نگردید که این خود نشان‌دهنده عدم ارتباط بین وقوع جهش در ناحیه پروموتوری 23-تا 214+ در ژن AR با ناباروری زنان می‌باشد. بر طبق نتایج حاصل از این مطالعه تفاوت معنا‌داری بین دو گروه زنان مبتلا و گروه زنان سالم یافت نشد (5/0=P). بحث و نتیجه ­گیری: نتایج این پژوهش حاکی از عدم ارتباط جهش ناحیه پروموتوری 23- تا 214+ ژن AR با نا باروری زنان در جمعیت مورد مطالعه می‌باشد.

Title: مقایسه ویژگی‌های شخصیتی و رفتار خوردن در زنان با شاخص توده بدنی بالا و طبیعی

http://yafte.lums.ac.ir/article-1-2464-fa.html

مقدمه: شیوع چاقی در جهان به‌شدت رو به افزایش است و افراد را در معرض خطر بالایی از سلامت قرار می‌دهد. پژوهش در زمینه عوامل روان‌شناختی دخیل در ایجاد چاقی می‌تواند گام مؤثری جهت پیشگیری و درمان چاقی باشد. بر همین اساس در این مطالعه، به بررسی مقایسه‌ای ویژگی‌های شخصیتی و رفتار خوردن در زنان چاق و با وزن طبیعی پرداخته‌ایم. مواد و روش­ ها: مطالعه حاضر از نوع علی-مقایسه ای بوده، جامعه آماری شامل زنان مراجعه کننده به بیمارستان‌ها و کلینیک‌های تغذیه مناطق یک و دو شهر تهران و نیز زنان با وزن طبیعی ساکن در این مناطق بودند. از جامعه آماری نمونه‌ای 150 نفری (75 زن چاق و 75 زن با وزن طبیعی) با روش نمونه گیری در دسترس انتخاب شد و پرسشنامه‌های ویژگی‌های شخصیتی نئو (NEO-FFI) و رفتار خوردن (TFEQ) را تکمیل کردند. داده‌ها با روش آماری t مستقل مورد تجزیه و تحلیل قرار گرفت. یافته ­ها: نتایج نشان داد گشودگی به تجربه و خویشتن داری شناختی در افراد چاق به‌طور معناداری کمتر از افراد با وزن طبیعی است و برعکس خوردن هیجانی و خوردن کنترل نشده در افراد چاق به‌طور معنادار بیشتر از افراد با وزن طبیعی است. بحث و نتیجه­ گیری: گشودگی به تجربه، خویشتن داری شناختی، خوردن هیجانی و خوردن کنترل نشده می‌توانند نقش مهمی را در ایجاد و حفظ چاقی داشته باشند که نیازمند توجه بیشتر می‌باشند.

Title: اثر مصرف کوتاه‌مدت عصاره آبی کلاله زعفران بر میزان مالون دی آلدئید و سیستم آنتی‌اکسیدانی کبد موش‌های نر جوان پس از یک جلسه فعالیت حاد وامانده ساز

http://yafte.lums.ac.ir/article-1-2474-fa.html

مقدمه: با وجود اثرات مفید فعالیت‌های منظم ورزشی، فعالیت‌های حاد وامانده ساز باعث بروز آسیب اکسیداتیو در کبد می‌شود. هدف از تحقیق حاضر بررسی اثر یک‌هفته‌ای مصرف عصاره آبی کلاله زعفران بر میزان تغییرات مالون دی آلدئید (MDA) و آنزیم‌های آنتی‌اکسیدانی سوپراکسیداز دیسموتاز (SOD)، کاتالاز (CAT) و گلوتاتیون پراکسیداز (GPX) کبد موش‌های نر جوان پس از یک جلسه فعالیت حاد وامانده ساز بود. مواد و روش­ ها: 48 سر موش نر ویستار به‌صورت تصادفی به سه گروه 1. کنترل یک (حلال عصاره زعفران) ۲. کنترل دو (حلال عصاره زعفران+ تمرین) 3. تجربی (mg/kg 50 عصاره آبی زعفران+ تمرین) دسته‌بندی شدند (هر گروه شامل 16 سر موش). پس از یک هفته، نیمی از هر سه گروه بدون وامانده شدن و نیم دیگر موش‌های گروه کنترل ۲ و تجربی بلافاصله پس از وامانده کردن قربانی شدند و مقادیر SOD، CAT، GPX و MDA کبد آنها اندازه‌گیری شد. یافته ­ها: متعاقب فعالیت وامانده ساز میزان MDA و فعالیت آنزیم‌های SOD، CAT، GPX بافت کبد موش‌های گروه تجربی برخلاف گروه کنترل، تغییر معنی‌داری نیافت (05/0<P‌). بحث و نتیجه­ گیری: مصرف عصاره آبی کلاله زعفران باعث تقویت سیستم دفاعی آنتی‌اکسیدانی کبد موش‌های ویستار شده و از تغییرات معنی‌دار سیستم دفاع آنتی‌اکسیدانی آنزیمی و MDA بافت کبد درنتیجه فعالیت وامانده ساز جلوگیری می‌کند.

Title: بررسی و مقایسه اثرات ضد سرطانی عصاره متانولی پیاز یزدی (Allium Jesdianum) و پیاز تابستانه لرستانی (Nectaroscordeum Coelzi) بر روی رشد سلول های HeLa و K562

http://yafte.lums.ac.ir/article-1-2475-fa.html

مقدمه: سرطان یک بیماری پیچیده با تغییرات ژنتیکی می­ باشد. از جمله روش‌های متعددی که برای بهبود و پیشگیری این بیماری بکار می­رود شیمی درمانی است که با توجه به مقاومت دارویی، اثرات جانبی داروهای سنتزی و اثبات پتانسیل دارویی بالای گیاهان، هدف از این مطالعه بررسی اثرات سمیت دو گیاه پیاز تابستانه لرستانی و پیاز یزدی از دسته لیلیاسه و مقایسه اثرات آنها در برابر دو سلول سرطانی انسان از رده سلول خون K562 و گردنه رحم HeLa می ­باشد. مواد و روش ­ها: در یک مطالعه تجربی پس از تهیه عصاره متانولی دو گیاه پیاز تابستانه لرستانی و پیاز یزدی، اثر سمیت هر کدام بر روی رشد سلول­ های سرطانی انسان از نوع خونی K562 و گردنه رحم HeLa در مقایسه با داروی استاندارد سیکلوفسفامید در طی زمان­ های 24، 48 و 72 ساعت با غلظت از 25/31 تا 250 میکروگرم بر میلی­لیتر با استفاده از آزمون MTT مورد بررسی قرار گرفت. یافته ­ها: نتایج بیولوژیکی بدست آمده در محیط آزمایشگاهی حاکی از آن است که سمیت عصاره ­های متانولی گیاهان مطالعه شده در این تحقیق وابسته به دوز می ­باشد و افزایش غلظت موجب کاهش معنی­دار سلول­ های سرطانی می­ شود. بر اساس داده ­های بدست آمده بیشترین سمیت 48 ساعت پس از افزایش عصاره این گیاهان اتفاق می­ افتد. همچنین آزمون MTT نشان داد که عصاره متانولی پیاز تابستانه لرستانی اثر سمیت بیشتری در مقایسه با پیاز یزدی بر علیه سلول HeLa داشت. درصورتی‌که بر اساس این آزمون حساسیت سلول K562 به عصاره پیاز یزدی بیشتر از عصاره پیاز تابستانه بود (01/0P<). علاوه­براین، عصاره گیاهان سمیت بیشتری در مقابل سلول‌های مطالعه شده از داروی سیکلوفسفامید نشان داد. بحث و نتیجه­ گیری: با توجه به نتایج بدست آمده عصاره متانولی پیاز یزدی و پیاز تابستانه لرستانی در غلظت ­های به‌کار رفته دارای اثرات سمیت بر روی سلول­ های K562 و HeLa می­ باشد. لذا می­توان جهت مطالعات بیشتر در محیط بدن موجود زنده از عصاره این گیاهان استفاده نمود.

Title: بررسی علل گرایش به مصرف مواد مخدر و داروهای روان گردان از دیدگاه دانشجویان دانشگاه علوم پزشکی لرستان

http://yafte.lums.ac.ir/article-1-2478-fa.html

مقدمه: پژوهش حاضر با هدف شناسایی علل گرایش به مصرف مواد مخدر و داروهای روان‌گردان از دیدگاه دانشجویان دانشگاه علوم پزشکی لرستان انجام شده است. مواد و روش‌ها: روش پژوهش حاضر، توصیفی-پیمایشی از نوع هدف کاربردی می ­باشد. جامعه آماری تحقیق شامل کلیه دانشجویان دانشکده­ های زیرمجموعه دانشگاه علوم پزشکی لرستان در سال تحصیلی 1394-1393 می­ باشد. برای نمونه ­گیری با توجه به جدول مورگان، 335 نفر با استفاده از نمونه‌گیری تصادفی طبقه‌ای انتخاب گردید. تحلیل داده­ ها در دو بخش آمار توصیفی، شامل جدول فراوانی و نمودار و آمار استنباطی، با استفاده از آزمون t تک نمونه‌ای توسط نرم افزار 19 SPSS انجام شده است. یافته ­ها: نتایج فرضیات تحقیق حاکی از آن است که عوامل فردی و عوامل محیطی و بین فردی و عوامل اجتماعی بر گرایش دانشجویان به مصرف مواد مخدر، تأثیر دارد (01/0P<). با توجه به نتایج بدست آمده در میان عوامل فردی، نگرش مثبت به مواد مخدر 33/23% و افسردگی 8/20% و عدم تحمل شکست و ناکامی 98/18% بیشترین میانگین را داشتند و کنجکاوی و پرخاشگری با 58/13% کمترین میانگین را داشت. در عوامل بین فردی و محیطی عوامل مربوط به خانواده با میانگین 96/30% بیشترین میانگین را داشت و عوامل مربوط به مدرسه 67/17% کمترین میانگین را بدست آورد. همچنین در عوامل اجتماعی عدم دسترسی به سیستم­ های خدماتی، حمایتی و مشاوره‌ای با میانگین 27/34% بیشترین میانگین و بازار مواد مخدر در ایران با میانگین 67/20% کمترین اولویت را بدست آوردند. در بین عوامل ذکر شده عوامل فردی با میانگین 62/23% بالاترین تأثیر را داشت. بحث و نتیجه­ گیری: به نظر می‌رسد احتمالاً برگزاری برنامه‌های جامع آموزشی و بالا بردن آگاهی، غنی سازی اوقات فراغت، ایجاد رفاه و نشاط در افزایش شناخت دانشجویان و درنهایت پیشگیری از مصرف مواد مخدر مؤثر می‌باشند.

Title: تأثیر عصاره الکلی برگ زیتون بر سطح سرمی LH، FSH و تستوسترون به دنبال آسیب ایسکمی- خون‌رسانی مجدد کلیوی در موش صحرایی نر

http://yafte.lums.ac.ir/article-1-2479-fa.html

مقدمه: کاهش تستوسترون در نرها یکی از عوارض ایسکمی کلیوی است. برگ زیتون یک منبع قابل ملاحظه ترکیبات فنلی است که به لحاظ بیولوژیکی فعال هستند و ظرفیت آنتی اکسیدانی، ضد التهابی و قدرت پاک کنندگی رادیکالی بهتری دارند. هدف از این مطالعه بررسی تأثیر عصاره برگ زیتون (OLE) بر تغییرات سرمی گنادوتروپین‌ها و تستوسترون بود. مواد و روش ­ها: در این مطالعه تعداد 49 سر موش صحرایی نر نژاد ویستار به‌صورت تصادفی انتخاب و به 7 گروه تقسیم‌ شدند: 1) کنترل: حیوانات سالم دست نخورده ،2) ایسکمی- خون رسانی مجدد 1 ساعت، 3) ایسکمی- خون رسانی مجدد 1 ساعت + OLE، 4) ایسکمی- خون رسانی مجدد 2 ساعت، 5) ایسکمی- خون رسانی مجدد 2 ساعت + OLE، 6) ایسکمی- خون رسانی مجدد 24 ساعت، 7) ایسکمی- خون رسانی مجدد 24 ساعت+ OLE. حیوانات در گروه‌های 3،5 و 7 به مدت 28 روز قبل از ایسکمی عصاره برگ زیتون را به میزان mg/kg100 از راه خوراکی و از طریق گاواژ دریافت کردند. در پایان دوره، نمونه خون اخذ و سطح سرمی اوره، کراتینین، گنادوتروپین‌ها و تستوسترون اندازه گیری شد. یافته ­ها: نتایج نشان داد که غلظت سرمی اوره و کراتینین در گروه کنترل نسبت به سایر گروه‌ها به‌طور معنی داری پائین است. همچنین مشخص گردید که در گروه 6 در مقایسه با گروه 7 غلظت سرمی تستوسترون به‌طور معنی داری کاهش یافت (05/0>P). بحث و نتیجه­ گیری: این مطالعه نشان داد که تجویز خوراکی عصاره برگ زیتون در گروه ایسکمی- خون‌رسانی مجدد 24 ساعت توانست از کاهش سطح سرمی تستوسترون جلوگیری نماید.

Title: اثر یک دوره تمرینات ثبات مرکزی بر ظرفیت عملکردی و خستگی در بیماران مبتلا به مولتیپل اسکلروزیس

http://yafte.lums.ac.ir/article-1-2480-fa.html

مقدمه: مولتیپل اسکلروزیس یک بیماری مزمن پیشرونده سیستم اعصاب مرکزی است و از علائم نشانه­ های آن خستگی و کاهش ظرفیت عملکردی می­ باشد. هدف از پژوهش حاضر بررسی تأثیر یک دوره برنامه تمرینی ثبات مرکزی بر ظرفیت عملکردی و خستگی در بیماران مبتلا به مولتیپل اسکلروزیس بود. مواد و روش ­ها: پژوهش حاضر از نوع نیمه تجربی با طرح پیش ­آزمون- پس ­آزمون بود. آزمودنی­ ها با دامنه سنی 40-20 سال و با مقیاس ناتوانی جسمانی (EDSS) یک تا چهار و هدفمندانه و داوطلبانه انتخاب و به‌صورت تصادفی به گروه ­های تجربی و کنترل تقسیم شدند. تمرینات به مدت هشت هفته و سه جلسه در هفته انجام گرفت و مدت هر جلسه یک ساعت بود. از تست عملکردی رسیدن (FR) برای اندازه­ گیری ظرفیت عملکردی و از مقیاس شدت خستگی (FSS) برای ارزیابی خستگی استفاده شد. داده ­ها با آزمون t مستقل و وابسته در سطح معناداری 05/0 مورد تجزیه و تحلیل قرار گرفتند. یافته ­ها: نتایج نشان داد که تمرینات ثبات مرکزی منجر به افزایش معناداری در ظرفیت عملکردی و کاهش معناداری در شدت خستگی شد (05/0P≤). همچنین تفاوت معناداری بین ظرفیت عملکردی و شدت خستگی در پس ­آزمون بین دو گروه تجربی و کنترل مشاهده شد (05/0P≤). بحث و نتیجه­ گیری: بر اساس یافته ­های پژوهش، تمرینات ثبات مرکزی می­ تواند عاملی برای بهبود ظرفیت عملکردی و کاهش شدت خستگی در بیماران مبتلا به مولتیپل اسکلروزیس باشد. با توجه به این نتایج، متخصصان مربوطه می­ توانند از این تمرینات به‌عنوان یک مکمل در کنار درمان‌های دارویی برای این بیماران استفاده کنند.

Title: طراحی و سنجش روایی و پایایی پرسشنامه‌ای به‌منظور شناسایی عوامل تأثیرگذار بر رفتار تغذیه‌ای افراد مبتلا به سندرم متابولیک

http://yafte.lums.ac.ir/article-1-2481-fa.html

مقدمه: مطالعات بسیاری اثر رژیم غذایی را بر اجزای سندرم متابولیک نشان داده‌اند. مطابق تئوری عمل منطقی، نگرش و هنجارهای انتزاعی عواملی هستند که بر روی قصد فرد به انجام یک رفتار و نهایتاً انجام آن رفتار توسط فرد اثرگذارند. هدف این مطالعه طراحی پرسشنامه‌ای معتبر به‌منظور شناسایی عوامل تأثیرگذار بر رفتار تغذیه‌ای افراد مبتلا به سندرم متابولیک می‌باشد. مواد و روش ­ها: در این مطالعه از طریق مرور متون علمی، 6 بحث گروهی متمرکز و مصاحبه با اساتید متخصص، پرسشنامه‌ای بر اساس تئوری عمل منطقی طراحی شد. به‌منظور بررسی روایی از روش‌های اندازه‌گیری روایی محتوا و روایی صوری با پانل خبرگان 15 نفری استفاده گردید و برای تعیین پایایی نیز از ضریب آلفای کرونباخ استفاده شد. یافته ­ها: پرسشنامه‌ی اولیه شامل 100 آیتم بود که پس از بررسی روایی و پایایی، تعداد آیتم‌های پرسشنامه‌ی نهایی 46 سؤال به دست آمد. 17 سؤال در بخش نگرش تغذیه‌ای، 13 سؤال در بخش هنجار انتزاعی و 16 سؤال نیز در بخش قصد رفتاری قرار گرفتند. میانگین شاخص روایی محتوای پرسشنامه‌ی نهایی 92/0 و ضریب آلفای کرونباخ پرسشنامه‌ی نهایی 85/0 به دست آمد. بحث و نتیجه ­گیری: بر اساس نتایج این مطالعه پرسشنامه‌ی طراحی شده در این مطالعه از روایی و پایایی نسبی خوبی برخوردار است و می‌تواند به‌منظور شناسایی عوامل تأثیرگذار بر رفتار تغذیه‌ای افراد مبتلا به سندرم متابولیک بر اساس تئوری عمل منطقی مورد استفاده قرار گیرد.

Title: شناسایی مورفولوژیک و مولکولی آسپرژیلوس‌های جداشده از بیماران بر اساس تعیین توالی ژن بتا توبولین (β-tubulin)

http://yafte.lums.ac.ir/article-1-2483-fa.html

مقدمه: گونه‌های آسپرژیلوس عامل پاتوژن فرصت طلب در افراد دارای ضعف سیستم ایمنی می‌باشند. این قارچ‌ها از جنبه‌های مختلف بیماریزایی و توکسین زایی حائز اهمیت هستند. هدف مطالعه حاضر، ارزیابی ژن بتاتوبولین در تشخیص و تعیین گونه‌های بالینی آسپرژیلوس‌ به روش تعیین توالی، در مقایسه با خصوصیات مورفولوژیک (مانند شکل کونیدی‌ها در آزمایش مستقیم، شکل کلنی در کشت و تست‌های فیزیولوژیک) می‌باشد. مواد و روش ­ها: در این مطالعه 465 بیمار مراجعه کننده به آزمایشگاه شفای اصفهان، مورد بررسی قرار گرفتند. شناسایی مورفولوژیک و مولکولی نمونه‌ های بالینی، به ترتیب با استفاده از کشت بر روی محیط‌ های سابورو آگار، مالت اکسترکت آگار و چاپکس آگار، مشاهده میکروسکوپی نمونه‌ ها و تقویت ژن بتا توبولین و تعیین توالی ناحیه مذکور انجام گردید. سکانس‌ها از طریق مقایسه با اطلاعات موجود در بانک ژنی آنالیز شدند. یافته­ ها: از 465 مراجعه کننده مشکوک، 39 مورد بیمار (8%) شناسایی شد. بیشترین جدایه‌ها مربوط به گونه فلاووس (56%)، اوریزآ (20%) و فومیگاتوس (10%) بودند. 59% بیماران زن و 41% مرد بودند. بحث و نتیجه­ گیری: در مقایسه با تست‌های فنوتیپیک، تعیین توالی ژن بتا توبولین برای شناسایی جدایه‌های بالینی آسپرژیلوس در سطح گونه بسیار با ارزش است. جایگزینی روش‌های مولکولی با روش‌های فنوتیپیک و تشخیص دقیق میکروارگانیسم به‌منظور درمان مؤثر به محققان این عرصه توصیه می‌گردد.

Title: بررسی عوامل مرتبط با اضافه وزن و چاقی در دانش آموزان شهر خرم آباد در سال تحصیلی 1391-1390

http://yafte.lums.ac.ir/article-1-2484-fa.html

مقدمه: چاقی و اضافه وزن با فاکتورهای بیولوژیکی، عوامل فردی، اجتماعی، اقتصادی و فاکتورهای مختلف فرهنگی و رفتارهای تغذیه‌ای در ارتباط است. شناخت این عوامل می­ تواند راهنمایی در جهت مداخلات مرتبط با پیشگیری و کنترل چاقی در کودکان و نوجوانان باشد. مطالعه حاضر با هدف بررسی عوامل تعیین­ کننده اضافه وزن و چاقی در دانش ­آموزان شهر خرم ­آباد انجام شد. مواد و روش­ ها: در این مطالعه مقطعی،3387 دانش ­آموز 6 تا 19 ساله با روش نمونه ­گیری تلفیقی طبقه­ ای و خوشه ­ای چند مرحله ­ای انتخاب شدند. قد و وزن دانش­ آموزان اندازه­گیری شد. همچنین شاخص توده بدنی (BMI) محاسبه گردید. جهت تعیین شیوع، از شاخص BMI استفاده شد و صدک بالای 95، به‌عنوان «چاق» و بین 85 تا 95، به‌عنوان «دارای اضافه وزن» در نظر گرفته شدند. یافته­ ها: ارتباط اضافه وزن و چاقی در دانش­ آموزان با نوع مدرسه، بعد خانوار، تحصیلات والدین و درآمد خانواده بسیار معنادار بود (001/0>P). چاقی و اضافه وزن در مدارس غیرانتفاعی و در خانواده ­های با درآمد بیشتر، شیوع بیشتری داشت. در دانش ­آموزانی که والدین آن­ها تحصیلات بالاتر داشتند، شیوع اضافه وزن و چاقی بیشتر بود. بین اضافه وزن و چاقی با وضعیت بلوغ ارتباط معناداری دیده نشد. بحث و نتیجه­ گیری: اضافه وزن و چاقی در دانش آموزان مورد بررسی با برخی عوامل فردی و بیرونی ارتباط معناداری داشت. از این عوامل می‌توان به جنسیت، رتبه تولد، بعد خانوار و میزان تحصیلات والدین اشاره کرد.

Title: کارایی داکسی سایکلین در کاهش اختلالات ناشی از ایسکمی - خون رسانی مجدد تجربی یک طرفه بیضه در اسپرم‌های اپیدیدیمی موش

http://yafte.lums.ac.ir/article-1-2485-fa.html

مقدمه: ایسکمی- خون ­رسانی مجدد ناشی از پیچش بیضه می ­تواند موجبات آسیب هر دو بیضه را فراهم آورد. مطالعه حاضر جهت ارزیابی اثر داکسی­سایکلین بر روی اختلالات ناشی از ایسکمی- خون­رسانی مجدد تجربی یک­طرفه بیضه در اسپرم‌های اپیدیدیمی موش صورت پذیرفت. مواد و روش­ ها: این مطالعه بر روی پنج گروه مساوی شش­ تایی موش نر بالغ انجام گرفت. در دو گروه از حیوانات، متعاقب بیهوشی و خارج نمودن بیضه سمت چپ، عروق بند بیضه و مجرای دفران از یکدیگر جدا و با استفاده از پنس خون ­بند غیرتروماتیک، بخش عروقی بند بیضه به مدت 30 دقیقه مسدود گردید. یک گروه از گروه ­های فوق، روزانه 5/2 میلی­گرم به ازای هر کیلوگرم وزن بدن به‌صورت داخل صفاقی داروی داکسی­سایکلین را به مدت 3 روز از روز ایجاد ایسکمی- خون­ رسانی مجدد تجربی دریافت نمود. گروه شاهد، شم و گروهی که تنها داکسی­سایکلین دریافت می­ کرد نیز در نظر گرفته شد. خصوصیات اسپرم هر دو اپیدیدیم پس از 35 روز مورد ارزیابی قرار گرفتند. یافته ­ها: ایسکمی- خون­ رسانی مجدد یک­طرفه بیضه کاهش معنی­داری را در تعداد، میزان زنده­ مانی و قابلیت تحرک اسپرم‌های هر دو اپیدیدیم موجب گشت. همچنین، میزان وقوع آپوپتوز در اسپرم‌های هر دو اپیدیدیم و بروز ناهنجاری در اسپرم‌های اپیدیدیم همسو به شکل معنی­ داری متعاقب ایسکمی- خون­ رسانی مجدد یک­ طرفه بیضه افزایش یافت. تجویز داکسی­سایکلین بهبود قابل ­توجهی را در تغییرات منفی ناشی از ایسکمی- خون­ رسانی مجدد در فراسنجه­ های فوق موجب گردید. بحث و نتیجه­ گیری: به نظر می­رسد داکسی­سایکلین به‌صورت بالقوه می­ تواند در برابر عوارض تولیدمثلی طولانی مدت ایسکمی- خون­رسانی مجدد یک­ طرفه بیضه در موش واجد اثرات محافظتی باشد.

Title: بررسی علل ا صلی و زمینه‌ای مرگ و میر نوزادان بر اساس کدبندی بین‌المللی بیماری‌ها در بیمارستان ابوذر اهواز

http://yafte.lums.ac.ir/article-1-2488-fa.html

مقدمه: تأمین، حفظ و ارتقاء سطح سلامت نوزادان به‌عنوان یک گروه آسیب پذیر در خدمات بهداشتی درمانی جایگاه ویژه­ای دارد و بی‌تردید اولین قدم در راه کاهش میزان مرگ و میر و ارتقاء سطح سلامت آنها، شناسایی علل مرگ و میر می‌باشد. مواد و روش­ها: در این مطالعه گذشته نگر، علل اصلی مرگ نوزادان از پرونده‌های نوزادان فوت شده در بیمارستان ابوذر در سال 1390 استخراج گردید و سپس بر اساس کدبندی جدید بین‌المللی بیماری‌ها (International Coding of Disease version 10) طبقه بندی شد و اطلاعات بر حسب سن حاملگی، نوع زایمان، وزن تولد، جنس و سن بعد از تولد تجزیه و تحلیل گردید. یافته­ ها: پنج علت اصلی مرگ به‌طور کلی عبارت بودند از نارسی، سندرم دیسترس تنفسی، سپتی‌سمی، آسفکسی و خونریزی داخل جمجمه. همچنین معلوم گردید که علل مرگ در نوزادان با وزن تولد کم با نوزادان نارس مشابه بوده و عبارت بودند از سندرم دیسترس تنفسی، سپتی‌سمی، آسفکسی و خونریزی داخل جمجمه. در این مطالعه بین مرگ با سن حاملگی، نوع زایمان، سن بعد از تولد و وزن تولد رابطه معنی دار وجود داشت ولی بین مرگ با جنسیت رابطه معنی داری یافت نشد. بحث و نتیجه ­گیری: در حالی که عفونت‌ها به‌عنوان علت اصلی مرگ نوزادان رسیده در این مطالعه بود، علل مهم مرگ در نوزادان نارس و کم وزن مطالعه ما با سایر مطالعات تفاوت عمده‌ای نداشت. با توجه به کاربرد آسان ICD10 و کارآیی آن در تعیین علل اصلی و زمینه‌ای مرگ نوزادان، استفاده از این طبقه بندی در گواهی‌های فوت نوزادان توصیه می‌شود.
